# High burden of self-reported sexually transmitted infections among female sex workers in Togo in 2021

**DOI:** 10.3389/fpubh.2022.915029

**Published:** 2022-08-03

**Authors:** Alexandra M. Bitty-Anderson, Fifonsi A. Gbeasor-Komlanvi, Akila W. Bakoubayi, Martin K. Tchankoni, Arnold J. Sadio, Mounerou Salou, Claver A. Dagnra, Didier K. Ekouevi, Patrick A. Coffie

**Affiliations:** ^1^African Research Center in Epidemiology and Public Health (CARESP), Lomé, Togo; ^2^University of Bordeaux, National Institute for Health and Medical Research (INSERM) UMR 1219, Research Institute for Sustainable Development (IRD) EMR 271, Bordeaux Population Health Centre, Bordeaux, France; ^3^PAC-CI Research Center – ANRS Site Côte d'Ivoire, CHU de Treichville, Abidjan, Côte d'Ivoire; ^4^Public Health Department, Faculty of Health Sciences, University of Lomé, Lomé, Togo; ^5^Molecular Biology Laboratory, Fundamental Sciences Department, University of Lomé, Lomé, Togo; ^6^National HIV/AIDS, Viral Hepatitis and Sexually Transmitted Infections Program (PNLS/HV/IST), Lomé, Togo; ^7^Laboratory Services, University Hospital Campus (CHU Campus), Lomé, Togo; ^8^Medical Sciences Research and Formation Unit, Dermatology and Infectiology Department, University Félix Houphouët-Boigny, Abidjan, Côte d'Ivoire; ^9^University Hospital of Treichville, Infectious and Tropical Diseases Service, Abidjan, Côte d'Ivoire

**Keywords:** sexually transmitted infections (STI), female sex workers (FSW), key populations, sub-Saharan Africa, STI symptoms

## Abstract

**Background:**

Sexually Transmitted Infections (STI) remain a public health challenge, especially for Female Sex Workers (FSW) who are particularly vulnerable. In sub-Saharan Africa where the overlapping epidemics of STI and HIV are concentrated among key populations, epidemiological data are needed in order to better understand STI trends in this population. The aim of this study was to assess the prevalence of self-reported STI symptoms and their risk factors among FSW in Togo in 2021.

**Methods:**

A cross-sectional study was completed in June and July 2021 among FSW in two cities of Togo: in Lomé (capital city, south of Togo) and in Kara (north of Togo). A snowball sampling method was used and after consent, a standardized questionnaire was administered by trained research staff to collect information on STI. A multivariate logistic regression model was used to identify factors associated with self-reported STI.

**Results:**

A total of 447 FSW, 300 in Lomé and 147 in Kara participated in this study. Median age was 30 [IQR: 24–38] and STI symptoms in the previous 12 months were reported by 191 FSW [42.7%, 95% CI: (38.1–47.5)], among whom 116 (60.7%) sought medical care. The most common reported symptoms were abnormal vaginal discharge (*n* = 78; 67.2%) and vaginal itching (*n* = 68; 58.6%). Current STI symptoms (at the time of the survey) were reported by 88 FSW (19.7%). In multivariable logistic regression, self-reported STI symptoms in the previous 12 months was negatively associated with living in the Lomé and older age and positively associated with having more than 15 clients in the previous week and being victim of violence.

**Conclusions:**

Findings from this study reveal that the self-reported STI burden is relatively high among FSW Togo. Additional strategies are needed at the structural, medical, political and social levels to curb this trend and effectively aim at the elimination of STI epidemics by the year 2030.

## Introduction

Sexually Transmitted Infections (STIs) are an important public health challenge worldwide, with an estimated 357 million new cases of four curable STIs (chlamydia, gonorrhea, trichomoniasis and syphilis) among 15–49 year olds, 417 million cases of herpes simplex type 2 (HSV2), and 291 million cases of human papillomavirus (HPV) among women each year (1). In sub-Saharan Africa (SSA), the incidence of STI was estimated at 63 million per year making it one of the most affected area in the World, sharing an estimated 40% of the global STI burden (1–3).

The epidemics of STIs have considerable repercussions on morbidity, mortality and the overall quality of life and wellbeing of individuals. STIs are among the main causes of infertility worldwide, are responsible for fetal and neonatal deaths (300 000 fetal and neonatal deaths each year), could lead to pelvic inflammatory disease and cervical cancer with HPV infection responsible for 530 000 cases of cervical cancer and 264 000 cervical cancer deaths each year (1, 4). Furthermore, STIs could cause cellular changes preceding cancers, and are recognized for facilitating HIV transmission (1). As such, key populations and female sex workers (FSW) in particular are among the most vulnerable population due to the social, economic and structural challenges they face. In fact, HIV prevalence among FSW in SSA is estimated at 36.9% compared to a 13.5% prevalence among women of reproductive age (5). The burden of STIs in this population is similarly high in SSA: 50 to 66% of FSW typically have a curable STI at any one time, 10% have active genital ulcer, 30% have syphilis, more than 30% have gonorrhea and chlamydia and 60% have HSV2 (6). In Togo, most recent estimates indicate an overall prevalence of STI among FSW ranging from 4.2% for gonorrhea to 32.9% for HPV in 2017, based on biological testing (7, 8).

In Togo and in most countries of SSA and low-and-middle-income countries (LMIC), the syndromic management of STI is recommended and syndromic management flow charts and algorithms remain the standard of care for the prevention and control of STI (9). The establishment of an etiologic diagnosis of STI, though ideal, could be difficult and unfeasible in those settings for several reasons including the lack of established laboratories that could delay same-day diagnosis results treatment or the lack of qualified human resources. In those cases, as is the case in Togo, a symptom-based approach to STI treatment has been recommended and simplified algorithms for each symptom have been developed by the World Health Organization (WHO) since 1984 and regularly updated since (9). Hence, in Togo screening and treatment of STIs primarily rely on self-reported symptoms. Based on this strategy and directly addressing this first pillar of the WHO global health sector strategy on STI of providing an update on the trends on STI prevalence, the aim of this study was to document the prevalence of self-reported STI and their risk factors among FSW in the two main cities of Togo (1).

## Materials and methods

### Study design and setting

A cross-sectional study, using a snowball sampling method, was conducted among FSW in June and July 2021 in the two most populous cities of Togo: Lomé, the capital city and Kara. Togo is a country of West Africa, with a population of 7.6 million inhabitants in 2018, covering 57,000 square kilometers with an average density of 133 inhabitants per square kilometers, an infant mortality of 45.2/1,000 and an estimated life expectancy of 64.5 years old. Lomé is located on the coast of the Atlantic Ocean, in the far south-western corner of Togo. Kara, the second most populous city of the country is located 413 km north of the capital city. Based on previous mapping and size estimation studies among key populations including FSW in Togo (9), Lomé and Kara were selected as study sites.

### Sample size and study population

FSW were eligible to participate in the study if they were 18 years and older; self-identified as sex workers (defined as engaging in transactional sex at least in the past 6 months; having sex in exchange for money or goods with clients as opposed to emotional partners or boyfriends); and were in the possession of a recruitment coupon. The sample size was determined based on STI prevalence among FSW in Togo in 2017 hovering at 6% (7, 8). With a precision of 3%, a confidence interval of 95% and an assumption of 10% missing data/non-response rate, the estimated minimum sample size was 264.

Steps were taken prior to the beginning of the study to identify and establish, with leaders from local FSW non-governmental organizations, the “hotspots” for FSWs, including brothels and bars. A total of 10 sites were selected across Lomé and 03 sites in Kara. A snowball sampling method was used to identify potential participants who then recruited other participants until the required sample size was reached at each site. Leaders were identified in each location, contacted and recruited into the study. Each leader received recruitment coupons to recruit other peers. A total of 2 leaders of FSW, who were also the first “seeds” were required per site (identified “hotspots”). The leaders from each site collaborated with the research team to arrange a space within or near the site that would protect the privacy of each participant. Leaders were informed of the research process and notified prior to the arrival of the research team. At each site, the research team was accompanied by a peer educator from a community-based FSW NGO. Questionnaires were pre-tested among three randomly selected FSW in Kara and Lomé.

### Data collection

After verifying eligibility and obtaining written consent, trained research staff (medical students) administered a face-to-face structured questionnaire in participant's language of choice (French, Ewe, Kabyè or English) using Google Forms. Data were collected using a questionnaire adapted from the guidelines on repeated behavioral surveys in population at risk of HIV (10) and based on an extended literature review on the subject. The questionnaire included questions on six main themes: (1) socio-demographic characteristics; (2) sexual history (types of partners, condom use); (3) STI and other sexual and reproductive health history; (4) Pre-exposure Prophylaxis; and (5) violence. Each questionnaire took approximately twenty minutes to complete. No personal identifiers were collected and each participant was assigned an identification number.

In addition, participants presenting STI symptoms at the time of the survey were offered the opportunity to be treated on the spot by a medical professional, based on the syndromic approach and STI guidelines currently in vigor in Togo (9). Another written consent was obtained for this part of the study, for those accepting to be treated. Participants were requested to provide a telephone contact information for follow-up on their symptoms and future referral in case of symptoms persistence. Patients were informed that their information will be kept confidential. Each participant received male condoms, lubricants and an amount of XOF 2,000 (around 4 US dollars) for their participation in the study.

### Measurements

Based on the syndromic management of STI, self-reported STI symptoms were considered a proxy for possible STI (9–11). Hence, self-reported STI during the past 12 months was assessed using the question: “*During the past 12 months, did you have any STI symptoms?*”; Medical consultations for those STI symptoms was assessed using the question: “*During the past 12 months, did you have a consultation in a health care service for those STI symptoms*?”. For symptoms of STI, participants were asked: “*Which of the following symptoms did you have during the past 12 months?*”. Choices of answers included abnormal vaginal discharge (odorous and/or colored), vaginal itching, genital ulceration, pain while urinating, abdominal pain. For current symptoms, the question asked was: “*Do you currently have STI symptoms?*”. The definition of STI was explained to participants prior to the beginning of the survey. Each measure was thoroughly explained to participants in case they could not understand. STI treatment was based on the WHO guidelines for the management of symptomatic STI and currently implemented in Togo. This guidelines provides specific treatment options and algorithm for each STI syndrome (9).

### Statistical methods

Data was collected on Google Forms, which provided directly a Microsoft Excel output. Analyses were performed using STATA software (STATA™ 12.0 College Station, Texas, USA). Results of descriptive analysis were presented as median and interquartile range (IQR) for quantitative variables and frequency and proportions for categorical variables. The prevalence of STI, calculated by dividing the number of TS who had at least one episode of STI by the total number of TS, and its 95% Confidence interval (95% CI) were calculated. Univariate and multivariate logistic regression were performed with a stepwise-descending selection procedure to identify factors associated with STI-self-report in the previous 12 months. The selection of covariates for multivariate analysis was based on the univariate analyses with factors associated with a *p*-value <0.20. We deemed a *p*-value <0.05 as statically significant for all analyses. Associations in the regression model were expressed as adjusted odds ratio (aOR).

### Ethical considerations

This study was approved by the Bioethics committee for Health research of the Ministry of Health (no. 11/2020) in Togo. Prior to the beginning of the study, participants were told about study purpose, procedures, protection of privacy and confidentiality and provided written consent. No identifiers were collected, hence no information that could reveal their identity was entered.

## Results

### Sociodemographic characteristics and sexual history

A total of 447 FSW were recruited, 300 in Lomé and 147 in Kara. Median age was 30 years old [Interquartile range (IQR): 24–38] and 63.8 % (*n* = 285) were single. Most FSW had reached at least a secondary school level of education (*n* = 218; 48.8%) and had other income generating activities aside from sex work (*n* = 262; 58.6%), mainly in trade and services (hairdressing, tailoring, housemaid, bar hostess) (Table 1A).

**Table 1A:** Sociodemographic characteristics of female sex workers in Togo in 2021 (*N* = 447).

**Characteristic**	**Total** **(*n* = 447)**	**Lomé** **(*n* = 300)**	**Kara** **(*n* = 147)**
	***N*(%)**	***n*(%)**	***n*(%)**
**Age (years)**			
Median [IQR]*	30 (24–38)	30 (24–39)	29 (23–36)
<30	22148.5	14749.0	7450.3
≥30	22651.5	15351.0	7349.7
**Level of education**			
None	8819.7	7625.4	128.2
Primary	14131.5	10033.3	4127.9
Secondary	19443.4	10836.0	8658.5
University	245.4	165.3	85.4
**Marital status**			
Living with a husband/a partner	4610.3	3010.0	1610.9
Divorced/separated/widowed	11625.9	7926.3	3725.2
Single	28563.8	19163.7	9463.9
**Nationality**			
Togolese	30267.5	16254.0	14095.2
Nigerian	8418.8	7926.3	53.4
Ghanaian	419.2	4113.7	00.0
Other**	204.5	186.0	21.4
**Other income generating activities**			
Yes	26258.6	17859.3	8457.1
No	18541.4	12240.7	6342.9

* *IQR, Interquartile Range*.

** *Côte d'Ivoire, Mali, Bénin, Burkina Faso*.

### Sexual history

Median age at first sex was 18 [IQR: 16–20] and median age at first transactional sex was 23 [IQR: 19–31]. Participants had a median of six clients [IQR: 4–10] in the preceding 7 days in Lomé, while in Kara, they had a median of four clients [IQR: 2–6] with an overall median earning between XOF 10,000 (17.29 USD) and XOF 25,000 (43.22 USD) in those preceding 7 days (Table 1B).

**Table 1B:** Sexual history of female sex workers in Togo in 2021 (*N* = 447).

**Characteristic**	**Total** **(*n* = 447)**	**Lomé** **(*n* = 300)**	**Kara** **(*n* = 147)**
	***N* (%)**	***n* (%)**	***n* (%)**
**Age at first sex (years)**			
**Median [IQR]**	18 (16–20)	18 (16–20)	18 (16–20)
<18	20145.0	13545.0	6644.9
≥18	24655.0	16555.0	8155.1
**Age at first transactional sex, Median [IQR]**	23 (19–30)	24 (19–32)	22 (18–30)
**Number of years in sex work**			
**Median [IQR]**	5 (3–11)	5 (3–11)	5 (3–11)
<1	6213.9	4515.0	1711.6
Between 1 and 3	15634.9	10836.0	4832.6
>3	22951.2	14749.0	8255.8
**Number of clients in the previous 7 days**			
**Median [IQR]**	5 (3–10)	6 (4–10)	4 (2–6)
≤7	30067.1	17658.7	12484.4
Between 8 and 14	9521.3	8127.0	149.5
≥15	5211.6	4314.3	96.1
**Income earned in the last 7 days (XOF)** ^**§**^			
Less than 10,000	12828.6	6321.0	6544.2
10,000–25,000	18541.4	13444.7	5134.7
25,000–100,000	11325.3	9632.0	1711.6
More than 100,000	40.9	31.0	10.7
Does not know/would not like to answer	173.8	41.3	138.8
**Use of condom during last sexual intercourse with a client**			
Yes	42895.7	29096.7	13893.9
No	194.3	103.3	96.1
**Consistent condom use with a client** (use of condoms during last intercourse AND during last 30 days)			
Yes	38489.7	26588.3	11981.0
No	6310.3	3511.7	2819.0
**Condom accident (breakage or slippage)**			
Yes	8819.7	7123.7	1711.6
No	35980.3	22976.3	13088.4

§ *1 USD = 581.2 XOF and 1 € = 657 XOF*.

A total of 259 (57.5%) FSW confirmed having a non0paying partner (defined as a boyfriend; emotional partner as opposed to clients, paying partner), and only 32.0% (*n* = 83) indicated using condoms with those partners during the last sexual intercourse. In regards to the use of condoms with clients, 95.7% (*n* = 428) indicated using condoms with their clients during their last sexual intercourse, and 87.7% (*n* = 392) indicated using condoms systematically (every time) with their clients in the previous month. Nearly a fifth of the sample (19.7%; *n* = 88) indicated having experienced condom breakage or slippage in the previous month. Almost three-fourth of the sample (*n* = 313; 70.0%) indicated having done an HIV test in the past 12 months and 21 of them (6.7%) reported being HIV positive (Table 1B).

### Self-reported STI

A total of 191 [42.7%, 95% CI: (38.1–47.5)] participants reported having STI symptoms in the 12 months preceding the survey. Among them, 116 (60.7%) reported consulting a medical professional for these symptoms. The most common STI symptoms reported among them were abnormal vaginal discharge (*n* = 78; 67.2%) and vaginal itching (*n* = 68; 58.6%), both significantly higher in Kara than in Lomé. Conversely, genital ulcer was significantly higher in Lomé than in Kara (45.6 vs. 10.2% *p* < 0.001) (Table 2). The median number of reported symptoms per participant over the preceding year was 2 [IQR: 2–3], so was the median number of consultations in a health care service for those symptoms over the year [IQR: 1–3]. More than a quarter (*n* = 33; 28.4%) reported going more than twice for consultations in a health care service for those STI symptoms.

**Table 2:** Self-reported sexually transmitted infections symptoms during the previous 12 months among female sex workers in Togo in 2021 (*n* = 447).

	**Total** **(*****n*** = **447)**		**Lomé** **(*****n*** = **300)**		**Kara** **(*****n*** = **147)**		***P***
	***n***	**%**	***n***	**%**	***n***	**%**	
STI symptoms self-report	191	42.7	122	40.7	69	46.9	0.200
Medical check-up for STI symptoms (yes)	116	60.7	57	46.7	59	85.5	**<0.001**
**Self-reported STI symptoms**							
Abnormal vaginal discharge	78	67.2	33	57.9	45	76.3	**0.03**
Abdominal pain	50	43.1	21	36.8	29	49.2	0.181
Genital ulcer	32	27.6	26	45.6	6	10.2	**<0.001**
Pain while urinating	28	24.1	11	19.3	17	28.8	0.231
Vaginal itching	68	58.6	24	42.1	44	74.6	**<0.001**

The bold values are significant values.

**Figure 1:**
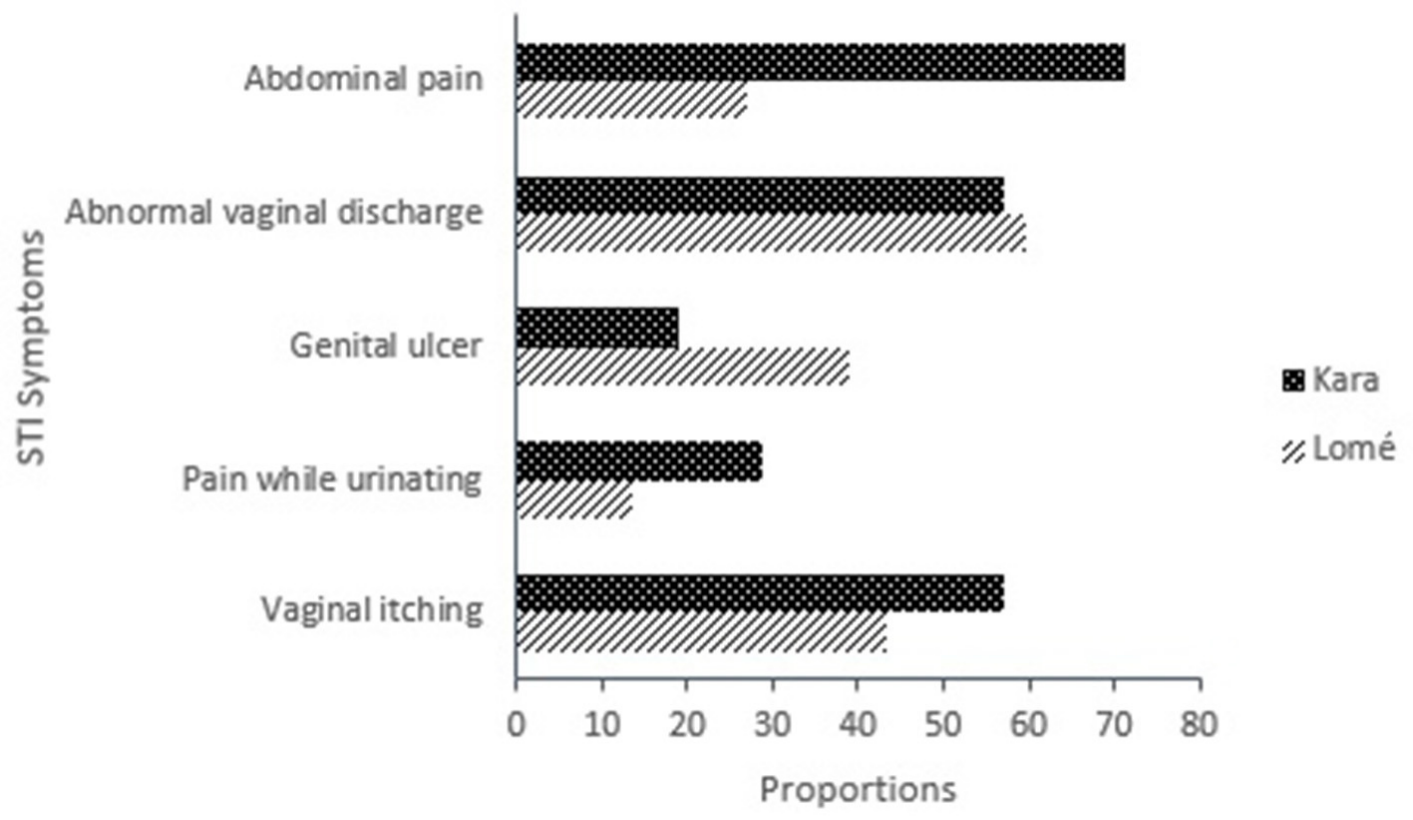
Self-reported current STI symptoms among FSW in Togo in 2021 (*n* = 88).

Eighty-eight participants [19.7%; 95% CI: (16.09–23.68)] reported current STI symptoms (at the time of the survey). The most commons symptoms reported at the time of the survey were abnormal vaginal discharge (*n* = 52; 59.1%) and vaginal itching (*n* = 41; 46.6%). Abnormal vaginal discharge was mostly reported by FSW in Lomé (*n* = 40; 59.7%); while abdominal pain was mostly reported in Kara (*n* = 15; 71.4%) with a significant difference between the two cities (*n* = 18; 26.9; *p* < 0.001) (Figure 1). The median number of current symptoms reported by each participant was 2 [IQR: (1–2)].

Fourteen women (15.9%) with current STI symptoms (at the time of the survey) indicated having consulted a medical professional for those symptoms. The 74 remaining women with at least one symptom at the time of the survey were treated according the WHO guidelines for the management of symptomatic STI, currently in vigor in Togo. A total of 31 FSW agreed to be contacted to document the disappearance or persistence of symptoms. Of these, 20 (64.5%) reported no symptoms after 10-days follow-up, two (6.5%) reported persisting symptoms (vaginal discharge and vaginal itching) and nine (29.0%) were lost to follow-up.

### Factors associated with self-reported STI

In multivariate logistic regression, living in Lomé [aOR = 0.56; 95% CI: (0.36–0.88, *p* = 0.01) and being 35 years old and older [aOR = 0.53; 95% CI: (0.31–0.90), *p* = 0.04] were negatively associated with self-reported STI symptoms. In addition, having more than 15 clients in the previous week [aOR = 2.30; 95% CI: (1.20–4.38), *p* = 0.02], and being a victim of violence (emotional, physical or sexual) [aOR = 1.61; 95% CI: (1.61–2.45), *p* = 0.03] increased the odds of self-reported STI symptoms (Table 3). Second analyses were performed using current STI self-report (*n* = 88) as dependent variable and no factors were associated with this variable.

**Table 3:** Factors associated with self-reported STI symptoms during the previous 12 months among FSW in Togo in 2021.

**Variables**	**Univariable analysis**		**Multivariable analysis**	
	**OR (95% CI)**	***p***	**aOR (95% CI)**	***p***
**City**				
Kara	1		1	
Lomé	0.77 (0.52–1.15)	0.20	0.62 (0.40–0.95)	**0.03**
**Age (years)**				
<25	1		1	
25–35	0.82 (0.52–1.29)	0.40	0.74 (0.46–1.19)	0.20
>35	0.57(0.34–0.93)	0.02	0.58 (0.35–0.97)	**0.04**
**Level of education**				
None/primary	1			
Secondary/university	0.89 (0.61–1.29)	0.54		
**Marital status**				
Married or living with a partner	1			
Divorced/separated/widowed	1.35 (0.67–2.71)	0.39		
Single	1.11 (0.58–2.10)	0.73		
**HIV testing in the previous 12 months**				
No	1			
Yes	0.81 (0.54–1.22)	0.32		
**Condom breakage**				
No	1			
Yes	1.52 (0.95–2.43)	0.07		
**Duration of sex work (years)**				
<1	1			
Between 1 and 3	1.00 (0.66–1.51)	0.99		
>3	1.12 (0.62–2.03)	0.70		
**Number of clients in the previous 7 days**				
≤7	1		1	
Between 8 and 14	1.29 (0.81–2.06)	0.27	1.32 (0.81–2.18)	0.24
≥15	2.30 (1.26–4.20)	0.006	2.15 (1.13–4.05)	**0.02**
**Use of condoms during last sexual intercourse with a partner**				
No	1			
Yes	0.59 (0.52–1.44)	0.59		
Non applicable (no partner)	0.86 (0.63–1.47)	0.86		
**Health care seeking for non-STI reasons**				
No	1			
Yes	0.67 (0.46–0.99)	0.04		
**Victim of violence (emotional, physical or sexual)**				
No	1		1	
Yes	1.61 (1.09–2.39)	0.01	1.60 (1.06–2.42)	**0.03**

The bold values are significant values.

## Discussion

This study sought to assess the prevalence of self-reported STI symptoms and its associated factors among FSW in the two main cities of Togo. The overall prevalence of STI self-reported symptoms over the previous 12 months was 42.7%, and among 60.7% of them who sought medical care for those symptoms, the most common STI symptoms were abnormal vaginal discharge (odorous and/or colored) and vaginal itching, despite quite high proportions of condom use with clients (95.7%).

This prevalence of self-reported STI symptoms in the previous year is high, but slightly comparable to those found in the region among key populations using the same method, with rates of 33.5% among FSW in Mozambique (11), and 35.6% in Ethiopia (12). Also, the current STI symptoms at the time of the survey was relatively high (19.7%), despite the self-reported high rate of condom use with clients during the last sexual intercourse (89.7%) and during the last 30 days (89.7%) in this population. Similar proportions of condom use have been observed in previous studies carried out in the region (13–15). Studies using biological methods for STI confirmation also confirm this high burden of STI among FSW with prevalence estimated at 43% for syphilis among HIV-positive FSW in Rwanda (16) and 35.1% of FSW having at least one STI in a study in Mali (17). In Togo, the highest prevalence found was that of hrHPV of 48.5% among HIV–positive FSW and 31.1% among HIV-negative FSW (7). The data on the incidence is rare in this population. In a survey conducted in Benin, incidence of STI was 10.8 event/person-years among FSW (18). All of these studies confirm high rate of STI among FSW. In comparison with data in the general population, studies among women indicate rates at least a third lower than that of FSW. For example, STI prevalence was assessed among women in the general population in Southern Africa, Southeast Africa and Eastern Africa and the overall prevalence of chlamydia, gonorrhea and syphilis among women aged 25 to 49 ranged from 0.9% to a maximum of 7.5% (19). Another study found a global prevalence of 4.2% for chlamydia, 0.8% for gonorrhea and 5.0% for trichomoniasis among women 15 to 49 years old (20). A better understanding of the dynamic of STI is needed in a context of high condom use among FSW, in order to develop more appropriate and adapted, tailored interventions for this population.

Among FSW who consulted for their STI symptoms, more than a third of the sample consulted more than twice throughout the year for STI symptoms, evoking either a STI reinfection or STI symptoms persistence. The high frequency of STI reinfection among FSW has been confirmed by other studies. In a study among Malagasy FSW, those who had an STI at baseline were 2–4 times more likely to have an STI during follow-up (21). In India, a study among FSW revealed high rates of STI persistence, despite an STI care package intervention (22). This study also demonstrated an increasing trend toward asymptomatic STI (22). This, coupled to the low accuracy of syndromic diagnosis of STI could lead to overtreatment, under treatment, missed treatment or incorrect treatment of STI (23). Advocacy could be directed toward additional effort for investment in simpler and more affordable laboratory diagnostics or the implementation of new and innovative strategies for STI diagnosis. Indeed, recent studies have suggested innovative strategies such as point-of-care tests (POCTS) under-development for the etiologic diagnosis of STI that could take into account the challenges and barriers faced in resource-limited settings and by FSW (3, 23). Other biomedical interventions include periodical antibiotic treatment or periodic presumptive treatment, although both methods have inconclusive results in terms of efficacy for STI prevention (3, 24, 25). Nevertheless, biomedical strategies should be combined with innovative behavioral and structural interventions that take into account the needs of this population (26). A paradigm-shift from strictly biomedical strategies for STI prevention among FSW to innovative public-health driven solutions that take into account structural, economic, social and political factors is an imperative (5, 27).

Several factors were associated with self-reported STI symptoms among FSW. FSW victim of violence were more than twice as likely to report STI. This result is consistent with previous studies that confirmed that violence is associated with sexual HIV risk and fear of accessing health care services (28). Similarly, a reduction of 25% in HIV incident cases was observed when physical and sexual violence was reduced in Ukraine and Kenya (29). Lifting barriers to access to care by providing stigma-free environment for this population, as well as addressing structural factors such as prevention of gender-based violence and the promotion of human rights should be a priority. The use of peer-educators and community-based organizations has been proven effective in the context of SSA (30). In addition to these, interventions comparing a differentiated model of care, tailored specifically to FSW and gearing only to them and an integrated model of care with trained healthcare professionals capable of catering to the specific needs of FSW, should be tested. Services that could be part of a differentiated model of care could include in addition to medical aspects of STI prevention, psychological and legal assistance. This study also found that self-reported STI symptoms was significantly associated with having more than 15 clients per week. Although a prevention message of limiting the number of clients would be difficult to achieve as this is related to income and thus livelihood of FSW, the consistent and correct use of condoms remains the only appropriate strategy. Condom use is an essential element of STI and HIV prevention with simulations indicating that condom use scale-up since 1990 has averted 117 million of new infections, half of them in SSA (31). However, promotion of condom use cannot be used as a stand-alone strategy as other factors such as access to condoms, condom use negotiation and economic and personal factors (i.e., partner's trust) influence the use of condoms in this population (15, 32). Combination prevention with access to condoms, education and continuous campaigns for raising awareness on the importance of the use of condoms with partners and clients, the involvement of partners and clients into STI prevention, are strategies that could be used to offset the burden of STI in this population (31). The involvement of partners in STI prevention strategies and as targets of STI prevention effort is of utmost importance, particularly since this study found that 32.0% of FSW who had a partner did not use condoms with them during their last sexual intercourse. Furthermore, older age was protective of STI self-reported symptoms. This finding should be further explored as the opposite trend has been observed in other studies, especially with HIV infection with older age associated with HIV infection (13, 16, 33). However, one hypothesis could be that young FSW lack the experience necessary to protect them against STI, such as inexperience in dealing with clients or in negotiating condom use (34). Young FSW face particular vulnerabilities putting them at heightened risk for STI and HIV, which should be specifically addressed in national strategies and policies of HIV and STI control.

In our study, some significant city differences were noted. FSW from Lomé had lower odds of STI self-reported symptoms compared to their counterparts in Kara. This finding could be expected when assuming the STI burden among FSW follows the same trend as the HIV pandemic in the same population. As such, previous data of HIV prevalence among FSW in Togo has indicated the highest prevalence of HIV among FSW in Kara (26.8%) (35). Despite higher vulnerabilities among FSW in Lomé (lower level of education, higher number of clients), more FSW in Lomé use condoms consistently with their clients compared to those in Kara which also could explain the lower odds of STI symptoms among FSW in Lomé. In addition, another hypothesis could be that FSW in Lomé, living in the capital with may have greater access to condoms in general and to sensitization campaigns on the importance of condom use with clients (36). There is a need for additional research on the STI prevalence across regions, and specifically in Kara to elucidate factors associated with high prevalence of STI and to develop targeted interventions specific to that region.

There are several limitations to this study. First, some symptoms declared might not directly be related to STI and in the same sense, asymptomatic STI might have been missed, which could have underestimated or overestimated the overall estimation of STI prevalence. Second a cross-sectional study was conducted, thus the temporal link between STI-self report and other factors could not be assessed. Also, as is the case with non-random sampling methods, the use of the snowball sampling method does not guarantee representation and could have introduced some bias. Finally, the use of a self-administered questionnaire could have generated biases, including social desirability bias (i.e., self-report of the use of condoms), memory bias and prevarication bias. Despite those limitations, this study is among the first in Togo to explore STI burden based on syndromic management and to show a relatively high prevalence of STI symptoms among FSW as demonstrated by other studies with STI diagnosis confirmed by laboratory analyses (17). Results from this study suggest a heavy burden of STI among FSW, and as such those findings could serve as the basis for the development of policies and actions toward a better management of STI among FSW, as a public health priority.

Further research should couple STI self-reported symptoms with confirmation of etiological diagnosis of STI among FSW, in order to assess the effectiveness of syndromic management of STI in this population. Although the syndromic approach to STI management has had a significant impact on the burden of STI over the years, a review of its continued performance should be considered, especially among most-at-risk populations, such as FSW. In addition, trials on STI interventions focusing on different methods for prevention of STI in this population should be done. Directly observed treatment (DOT) or field-delivered therapy for STI as experienced in this study seems an interesting approach that has proven to be successful for the management of STI (37). However, the results observed should be confirmed. To our knowledge limited data are available using this approach for vulnerable populations, specifically FSW and for STI treatment, for comparison and lessons learned. Qualitative studies should also explore more deeply barriers to access to care and reasons for non-consultation among FSW, as solutions to reduce the gap between FSW and utilization of health care services should be a priority.

## Conclusion

Self-reported STI is highly prevalent among FSW, with most common symptoms abnormal vaginal discharge and vaginal itching and risk factors for STI symptoms including a high number of clients (more than 15) and being victim of violence. On the other hand, living in the capital city and being older (35 years old and older) were protective of self-reported STI. Additional research is needed in the area of STI prevention, treatment and management among FSW, including improvements in diagnosis, innovative interventions such as directly-observed-treatment as experimented in this study. Policy advocacy for structural improvements taking into account gender-based violence and access to care could also contribute to reducing the burden of STI and HIV among FSW in Togo.

## Data availability statement

The original contributions presented in the study are included in the article/supplementary material, further inquiries can be directed to the corresponding author/s.

## Ethics statement

The studies involving human participants were reviewed and approved by Bioethics Committee for Health Research, Ministry of Health, Togo (No. 11/2020). The patients/participants provided their written informed consent to participate in this study.

## Author contributions

PC, DE, AB-A, and FG-K conceived and planned the study. AB-A, AB, AS, MS, and CD implemented the study and collected the data. AB-A, MT, and AS analyzed the data. AB-A wrote the original draft with input from PC, DE, and FG-K. All authors reviewed the results and approved the final version of the manuscript.

## Funding

This work was supported by the African Research Center on Epidemiology and Public Health (CARESP).

## Conflict of interest

The authors declare that the research was conducted in the absence of any commercial or financial relationships that could be construed as a potential conflict of interest.

## Publisher's note

All claims expressed in this article are solely those of the authors and do not necessarily represent those of their affiliated organizations, or those of the publisher, the editors and the reviewers. Any product that may be evaluated in this article, or claim that may be made by its manufacturer, is not guaranteed or endorsed by the publisher.
